# Genome-Wide Association Study and Transcriptome of Japanese Patients with Developmental Dysplasia of the Hip Demonstrates an Association with the Ferroptosis Signaling Pathway

**DOI:** 10.3390/ijms24055019

**Published:** 2023-03-06

**Authors:** Yu Mori, Kazuko Ueno, Daisuke Chiba, Ko Hashimoto, Yosuke Kawai, Kazuyoshi Baba, Hidetatsu Tanaka, Takashi Aki, Masanori Ogasawara, Naoto Shibasaki, Katsushi Tokunaga, Toshimi Aizawa, Masao Nagasaki

**Affiliations:** 1 Department of Orthopaedic Surgery, Tohoku University Graduate School of Medicine, Sendai 980-8575, Japan; 2 Genome Medical Science Project, National Center for Global Health and Medicine, Tokyo 162-8655, Japan; 3 Human Biosciences Unit for the Top Global Course Center for the Promotion of Interdisciplinary Education and Research, Kyoto University, Kyoto 606-8507, Japan

**Keywords:** cartilage, developmental dysplasia of the hip, ferroptosis signaling pathway, genome-wide association study, Japonica array, psychiatric disorders, UK Biobank

## Abstract

This study examined the association between developmental dysplasia of the hip (DDH) and disease-associated loci in a Japanese cohort. A genome-wide association study (GWAS) of 238 Japanese patients with DDH and 2044 healthy individuals was performed. As a replicate, GWAS was also conducted on the UK Biobank data with 3315 cases and matched 74,038 controls. Gene set enrichment analyses (GSEAs) of both the genetics and transcriptome of DDH were performed. Transcriptome analysis of cartilage specimens from DDH-associated osteoarthritis and femoral neck fractures was performed as a control. Most of the lead variants were very low-frequency ones in the UK, and variants in the Japanese GWAS could not be replicated with the UK GWAS. We assigned DDH-related candidate variants to 42 and 81 genes from the Japanese and UK GWASs, respectively, using functional mapping and annotation. GSEA of gene ontology, disease ontology, and canonical pathways identified the most enriched pathway to be the ferroptosis signaling pathway, both in the Japanese gene set as well as the Japanese and UK merged set. Transcriptome GSEA also identified significant downregulation of genes in the ferroptosis signaling pathway. Thus, the ferroptosis signaling pathway may be associated with the pathogenic mechanism of DDH.

## 1. Introduction

Osteoarthritis (OA) of the hip causes pain and disability in elderly individuals. Developmental dysplasia of the hip (DDH) is one of the most important causes of OA [1,2,3,4]. The mean incidence of DDH varies widely according to ethnic background, with rates of 0.06, 76.1, and 3.6 per 1000 live births in Black African, Native American, and British populations, respectively [5]. However, epidemiological studies of radiographic imaging of DDH in the Japanese population show that the rate of radiological DDH (center–edge angle less than 25° [6]), including asymptomatic DDH, is 16% and 19% [7], and 5.1% and 11.6% in men and women, respectively [8]. In particular, the incidence of DDH has been reported to be higher in the Japanese population than in other ethnic groups. DDH is a complex disorder with known associations with the female sex, first birth, breech birth, and family history [9]. There is no causative therapy for DDH, and hip replacement is performed when the joint destruction progresses. The reported benefits of hip arthroplasty in easing pain and improving function are widespread [10,11]. However, there is a need for revision surgery due to loosening, and although efforts have been made to prevent stress shielding with low-modulus titanium alloys [12], there is room for improvement.

DDH is heritable; however, its genetic association has not yet been fully elucidated. There have been reports of genetic polymorphisms in the OA-related genes growth differential factor 5 (*GDF5*) [13,14], calmodulin 1 (*CALM1*) [15,16], and asporin [17]. In contrast, there have been several reports on DDH-related genes. Several linkage scans and candidate gene studies have suggested the possibility of related genetic variants, including *GDF5* [9,18,19,20]; however, to date, only a few studies have identified a genome-wide significant locus [21,22,23]. The C-X3-C motif chemokine receptor 1 (*CX3CR1*) gene has also been associated with DDH, and abnormal hip morphology has been reported in mice deficient in this gene [24,25,26]. A genome-wide association study (GWAS) in the Han Chinese population suggested an association between mutations in the ubiquinol–cytochrome C reductase complex assembly factor (*UQCC*)—a gene adjacent to *GDF5* [27]. However, no DDH-sensitive gene has been reported in the Japanese population, which experiences a high prevalence of this disease.

The GWAS tests millions of gene variants across the genomes of many individuals to identify genotype–phenotype associations. Since the first GWAS for age-related macular degeneration was published in 2005, a number of reports have been published [28,29].

The Tohoku Medical Megabank Organization constructed a reference panel containing over 20 million single nucleotide polymorphisms (SNPs) from the whole-genome sequence data of 1070 Japanese general individuals [30]. A new custom-made genotyping SNP array with approximately 659,253 SNPs, named Japonica array v1, which was optimized to impute the non-observed variants from the genotyping SNPs, especially for Japanese individuals, was applied [31]. The Japonica array v1 has GWAS experience for risk gene exploration in glaucoma [32], osteoporosis among patients with inflammatory bowel disease [33], and primary biliary cholangitis [34] in Japanese patients. Based on this evidence, we considered it reasonable to analyze risk factors for DDH using Japonica array v1, which is the most appropriate GWAS for the Japanese population.

The purpose of this study was to examine the association between DDH and disease-associated loci in a Japanese population and to assess the genotype–phenotype relationships between risk variants and clinical features of the disease.

## 2. Results

### 2.1. Demographics and Clinical Data

The characteristics of 238 Japanese patients with DDH are shown in Table 1. Of the 238 patients, 206 (86.5%) were female.

The mean age at the time of genomic DNA collection was 60.0 ± 15.0 years, and 94 patients (39.4%) had a family history of DDH. A history of orthotic or casting treatment for DDH during childhood was detected in 110 cases (46.2%), and surgical treatment for DDH was performed in 161 cases (67.6%). Seventy-two (30.0%) patients underwent hip replacement surgery. Transcriptome analysis of chondrocytes was performed in 12 cases of DDH-related OA and 8 cases of femoral neck fracture; 11 out of the 12 patients with DDH-related OA were female, and the mean age at the time of surgery was 64 ± 7 years. Seven of the eight patients with femoral neck fractures (control) were women, and the mean age at the time of surgery was 80 ± 7 years. Qualitative real-time polymerase chain reaction (RT-PCR) was performed in 14 cases of DDH-related OA and 14 cases of femoral neck fracture; 13 of the 14 patients with DDH-related OA were female, and the mean age at the time of surgery was 69 ± 5 years. In addition, 11 of the 14 patients with femoral neck fractures (control) were women, and the mean age at the surgery was 80 ± 13 years.

### 2.2. Genome-Wide Association Analyses of DDH in Japanese Patients

**Figure 1:**
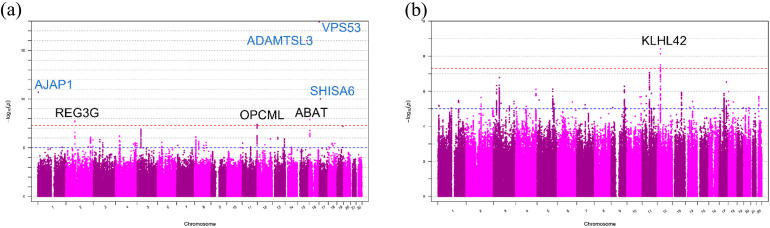
Manhattan plots of association of SNPs in Japanese patients with DDH and the UK Biobank data set. (**a**) Manhattan plot of the Japanese case and control studies with DDH. The gene in blue denotes the result from the direct genotyping of SNPs. (**b**) Manhattan plot of the UK Biobank case and control studies with DDH. The broken red line indicates a *p*-value < 5 × 10^−8^. The broken blue line indicates a *p*-value < 1 × 10^−5^. DDH, developmental dysplasia of the hip; SNP, single nucleotide polymorphism.

After quality control (QC; Table 2) of the genotyping results of 238 DDH samples and 2044 samples from the general population in Japan, the whole-genome genotype imputation process was applied using the phased reference panel of Phase 1 version 3 of the 1000 G containing 1092 individuals [35]. For the imputed genotyping results, a GWAS was conducted (referred to as the *“*Japanese GWAS*”*). Four directly genotyped SNPs in different locations reached the genome-wide significance level (*p* < 5 × 10^8^): rs11802858 (chr1:4682515:T:C; 1.98 × 10^−11^); rs2554380 (chr15:84315884:C:T; 1.04 × 10^−16^); rs79657649 (chr17:505105:T:C; 1.19 × 10^−18^); and rs17699467 (chr17:11359275:A:G; 9.82 × 10^−11^) (Table 2a, Figure 1a).

The genotyping concordance rates of all four direct genotyping SNPs were high (>0.98) between the genotyping results of the SNP array and the whole-genome sequence data for the shared 190-sample set (Table S1). Notably, there were no SNPs with high linkage disequilibrium (LD) (r^2^ > 0.2) with rs79657649 around this SNP. rs2554380 is positioned in the promoter region of *ADAMTSL3* and is related to many phenotypes in a phenome-wide association study (PheWAS), such as standing height and impedance of the leg (Table S1). rs79657649 is located in the coding region of *VPS53,* and the minor allele of this SNP causes a missense mutation (Asn > Ser). rs17699467 is present in the intronic region of *SHISA6*. 

After genotype imputation, no additional variants reached the significance level of the former four regions, while three additional loci reached the genome-wide significance level (*p*-value < 5.0 × 10^−8^; Table 2); the three lead variants in each locus were rs11802858 (chr1:4682515:T:C; 1.98 × 10^−11^) in the upstream (around 32 kb) from the transcription start site (TSS) of *AJAP1*, rs149003127 (chr2:79236134:G:T; 1.82 × 10^−8^) in the upstream (around 16 kb) from the TSS site of *REG3G*, rs55669018 (chr11:132871263:T:A; 4.09 × 10^−8^) in the intronic region of *OPCML*, and rs77485026 (chr16:8846734:T:C; 1.34 × 10^−8^) in the intronic region of *ABAT* (Figure 1a).

### 2.3. Replication Study and Genome-Wide Association Analyses of DDH in the UK Biobank Data Set

To conduct the replication study as an independent cohort study, part of the participants in the UK Biobank with White British genetics were used. In this study, 3315 samples assigned to at least one category (M161, M162, and M163 in ICD10) were selected as DDH patients, and 74,038 healthy individuals were selected as controls.

The minor allele frequencies (MAF*s*) of rs79657649 and rs11802858 were lower than 0.01 in the UK population (0.001618/6 [MAF/allele count] and 0.000809/3, respectively, in TwinsUK [36]) and could not be included as replication targets. For the remaining two SNPs, the genotyping information of rs2554380 and rs17699467 (0.22411/831 and 0.141046/523 in TwinsUK*,* respectively) was reported in the imputed genotyping result in the UK Biobank and the nominal *p*-values were 0.63 and 0.098, respectively. *F*or rs17699467, in a PheWAS of the Finnish population, a phenotype “disease of the musculoskeletal system and connective tissue” reached the phenome-wide significance level in the data freeze 5 *(*https://r5.finngen.fi/ (accessed on 1 August 2022.); Bonferroni *p*-value threshold *=* 1.78 × 10^−5^ [0.05/2803]; *p* = 9.4 × 10^−6^; Figure S1).

The MAF*s* of rs149003127 and rs77485026, which reached the genome-wide significance level in the Japanese GWAS after imputation, were lower than 0.01 in the UK population and could not be included as replication targets. Another SNP, rs55669018, which also reached the genome-wide significance level in the Japanese GWAS after imputation, had a nominal *p*-value of 0.81 and could not be replicated in the UK dataset.

For the downstream analysis, a GWAS was conducted (UK GWAS) on the UK dataset with case and control, and one locus reached the genome-wide significance level (*p* = 3.84 × 10^−8^) (Table 2b and Figure 1b).

### 2.4. Gene Mapping of Disease-Related Variants and Gene Set Enrichment Analysis (GSEA)

In previous DDH studies [9,18,19,20,21,22,23,24,25,26,27], a very limited number of shared genetic variants was detected among different studies. In our study, most of the discovered candidate variants could not be directly replicated because of the different allele frequencies or LD between the Japanese and White British populations. 

Thus, we attempted to interpret the DDH disease-related factors from the categories of variant-related genes. For gene-based enrichment analysis, we extracted suggestive SNPs (*p*-value < 1 × 10^−5^) for downstream analysis. Using functional mapping and annotation (FUMA), 42 and 96 genes were assigned to the Japanese GWAS and UK GWAS, respectively (Tables S2 and S3). There were no shared genes between the two cohort studies.

Using each gene set, we performed three functional enrichment analyses using FUMA2FUNC, ingenuity pathway analysis (IPA), and ToppGene (https://toppgene.cchmc.org/enrichment.jsp (accessed on 1 August 2022) [37]). For both cohorts, no significant pathway enrichment (adj. *p* < 0.05) was observed in the FUMA2FUNC analysis.

In the IPA, no significant category was enriched in the UK gene set; however, one significantly enriched category was found in the Japanese gene set (Table 3a). Pathway gene sets were considered significantly enriched when the Benjamini–Hochberg (BH) false discovery rate (FDR) was <0.1, and there were at least three overlapping genes between the gene set and the study gene list. The enriched category was the ferroptosis signaling pathway (*p*-value = 0.0015; BH FDR 0.065), with three DDH-related genes: cathepsin B (*CTSB*; chr8:11,698,033-11,727,596), farnesyl-diphosphate farnesyltransferase 1 (*FDFT1*; chr8:11,651,082-11,698,818), and GTP cyclohydrolase-1 (*GCH1*; chr14:55,306,723-55,371,542). Ferroptosis is a type of programmed cell death, which was predicted to be related to OA in a previous study [38].

In the ToppGene disease analysis, no category was significantly enriched in the Japanese gene set. Pathway gene sets were considered enriched when the Bonferroni *p*-value was < 0.01, and there were at least three overlapping genes between the gene sets. However, two categories were significantly enriched in the UK GWAS (Table 3b). The top hit was OA, including four DDH GWAS-related genes on different chromosomes: *ITIH1* (chr3:52,809,615-52,828,078), *ASTN2* (chr9:119,183,391-120,179,335), *LTBP3* (chr11:65,304,030-65,327,699), and *IL11* (chr19:55,873,750-55,883,831). The second most common hit was bipolar disorder. An epidemiological study was conducted on 74,393 patients with psychiatric disorders to assess the association between psychiatric disorders and OA. The study reported that affective psychoses, neurotic illnesses, personality disorders, and other mental disorders are risk factors for OA [39]. Thus, there might be genetic risk factors, such as *ITIH1*, between the top-hit OA and the second-hit mental disorder.

### 2.5. Pathway Enrichment Analysis for the Merged Gene Set

To interpret the shared DDH disease factors among the two studies with different genetic backgrounds, the 42 and 81 Japanese and UK GWAS genes were merged into 123 genes and re-analyzed using the same tools, FUMA2FUNC, IPA, and ToppGene.

In the FUMA2FUNC analysis, there were two significant categories (the condition is the same as the definition in 2.3), cytoskeleton organization (Gene Ontology [GO]: 0007010) and cell signaling (GO: 0007267) (Table 3c). The GO category cytoskeleton organization contained seven genes from the Japanese GWAS and nine genes from the UK GWAS. Notably, three dynein axonemal heavy chain (DNAH) family members in different chromosomes were enriched in DDH: *DNAH1* (chr3:52,348,335-52,436,508); *DNAH7* (chr2:196,600,427-196,935,561); and *DNAH8* (chr6:38,681,087-39,000,568). *DNAH1* was a candidate gene in the UK GWAS, and *DNAH7* and *DNAH8* were candidate genes in the Japanese GWAS.

In the IPA, the ferroptosis signaling pathway was found to be the top *p*-value pathway by adding a novel gene from the UK GWAS, BRCA1 associated protein 1 (*BAP1*; chr3:52,433,024-52,446,024) (Table 3d).

In ToppGene disease analysis, OA was still the top *p*-value pathway (Table 3e). However, none of the DDH risk genes in the Japanese population were categorized as OA. In contrast, bipolar disorder, which was significant in the UK GWAS dataset, was also significantly present in the merged set by adding two novel genes from the Japanese GWAS: *DNAH8* (chr6:38,681,087-39,000,568) and *IMPA1* (chr8:82,567,151-82,600,589). This result further suggests a potential general risk association between mental illness and DDH.

### 2.6. Transcriptome Analyses of OA Associated with DDH in Japanese Patients

**Figure 2:**
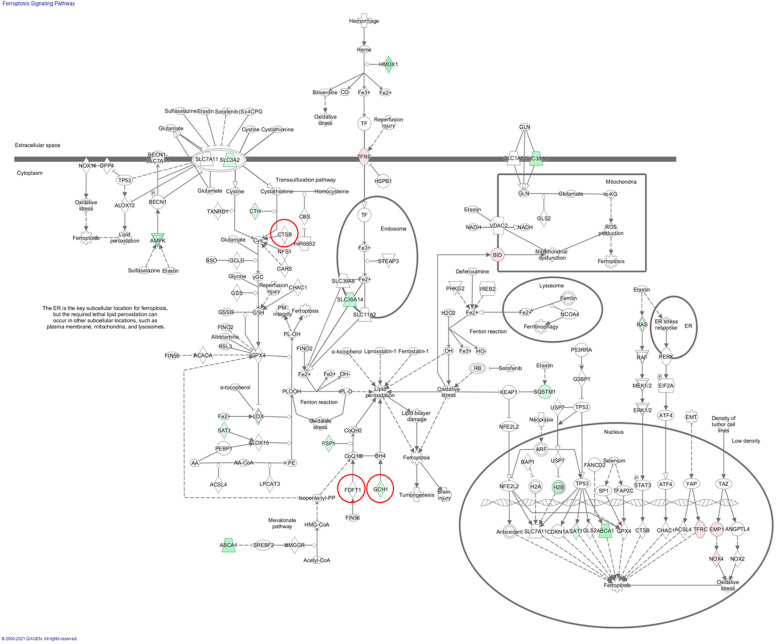
Ferroptosis signaling pathway using IPA. The three red circled genes are listed in the genes from the Japanese GWAS. The genes with pink background are upregulated in OA, and those with green background are downregulated in OA. IPA: ingenuity pathway analysis; GWAS: genome-wide association study; OA: osteoarthritis.

**Figure 3:**
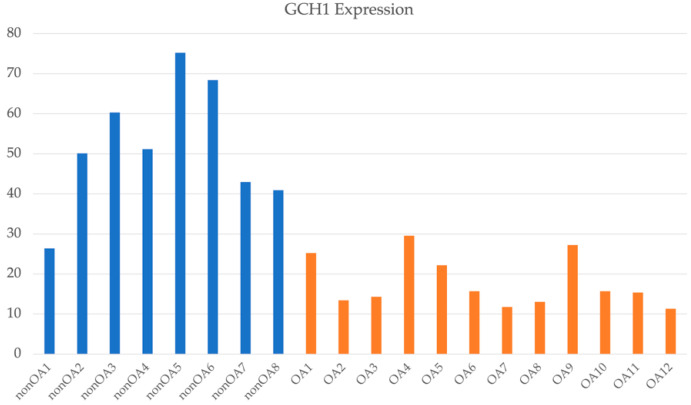
*GCH1* expression in samples from 8 non-OA patients and 12 OA patients. OA: osteoarthritis.

In all, 1079 and 822 genes were significantly expressed in DDH-related OA patients (DDH patients) compared to that in the control samples. Related pathways were predicted for each gene set using the IPA. The GP6 signaling pathway was at the top of the former gene set (*p* = 3.98 × 10^−17^), and the ferroptosis signaling pathway was at the top of the latter gene set (*p* = 1.67 × 10^−7^). As predicted by the Japanese GWAS, the ferroptosis signaling pathway was independently predicted by transcriptome analysis from the Japanese dataset. Seventeen genes have been identified in this pathway. We analyzed the shared genes categorized in this pathway from both the genomic and transcriptomic perspectives (Figure 2). *GCH1* is a disease susceptibility gene in the Japanese GWAS and was expressed significantly less in DDH samples than in healthy individuals (Figure 3). This indicates that a lower gene expression level than that in the control group is possibly associated with the disease.

### 2.7. Expression Analysis of GCH1 Using Qualitative Real-Time Polymerase Chain Reaction (RT-PCR)

**Figure 4:**
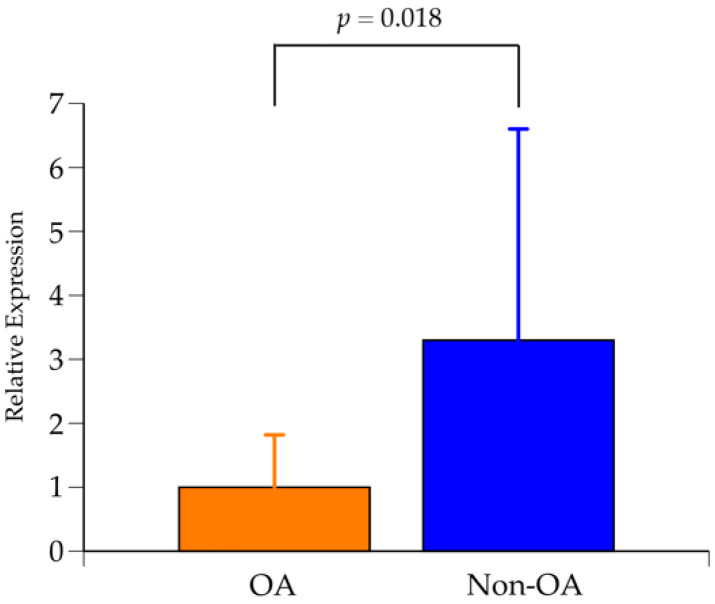
*GCH1* expression in 14 non-OA patients and 14 OA patients by qualitative real-time polymerase chain reaction. OA: osteoarthritis. *p* = 0.018 by Student’s *t*-test.

The results of qualitative RT-PCR for the expression of *GCH1* are shown in Figure 4. *GCH1* expression in the DDH-related OA group had significantly decreased compared to the non-OA group. RT-PCR analysis was performed using chondrocyte-derived RNA from different cases from the transcriptome analysis; RT-PCR results were able to reproduce the results of the transcriptome analysis.

## 3. Discussion

In the present study, a GWAS of 238 Japanese patients with DDH and 2044 patients from the general population was performed. Several significant SNPs were found, none of which had been previously reported; thus, the authors referred to the UK Biobank data. As there were few significant SNPs, we extracted suggestive SNPs with *p* < 1e-05. We then assigned genes to those SNPs: 81 genes from the GWAS of the UK Biobank dataset and 42 genes from the GWAS of the Japanese patients using FUMA. No similarities were observed between the two gene sets. Ontology analysis of each gene set using FUMA and IPA revealed no significant pathways in the UK Biobank gene set; however, significant pathways were identified as culminating in the ferroptosis signaling pathway in the gene set of the Japanese patients. This pathway is associated with OA [40,41,42]. It has also been reported that the ferroptosis signaling pathway is present in the chondrocytes of OA patients [43]. However, no studies have demonstrated that OA progresses due to the inhibition of ferroptosis signaling using clinical specimens from patients with OA. The current study is the first to report an association between the ferroptosis signaling pathway and DDH.

In a comparative analysis of chondrocyte transcriptomes of patients who underwent arthroplasty for secondary hip OA after DDH and those with femoral fractures without articular cartilage degeneration, the expression of *GCH1, CTSB,* and *FDFT1*, which are factors in the ferroptosis signaling pathway, was lower in the hip OA group than in the control group. *GCH1* has been reported to be associated with ferroptosis in esophageal and colorectal cancers [44,45]. *CTSB* and *FDFT1* are associated with ferroptosis in pancreatic and kidney cancer, respectively [46,47]. Transcriptome analysis of OA chondrocytes showed that *GCH1*, a component of this pathway, was significantly downregulated. In a future study, we will identify the genes involved in this pathway using IPA and confirm the expression variation of these genes. The importance of genes involved in the ferroptosis signaling pathway in OA is not clear, but there may be differences between OA and DDH-related genes such as *GCH1, CTSB,* and *FDFT1*. As the OA cases analyzed in this study were DDH-related, transcriptome analysis of chondrocytes confirmed that the variation in ferroptosis signaling in these cells was significant. It was also interesting to note that the results of GWAS and chondrocyte transcriptome analysis showed a consistent trend.

The GDF5-UQCC1 region is frequently reported as the related region to DDH. In this region, our Japanese GWAS had a consistent feature to previous DDH studies [21,27,48]. In a Chinese population study [48], rs143384 was reported as the significant lead variant. In our Japanese population GWAS, the rs143384 had the *p*-value 0.0029 with the same direction OR 1.50. In our GDF5-UQCC1 region, the lead *p*-value SNP was rs34414056 (OR = 2.06, *p*-value = 0.000554). This SNP is located in the intronic region in GDF5 (Figure S2). For the rs34414056, a Japanese eQTL database ImmuNexUT reported UQCC1, and a European eQTL GTEx V8 database reported both GDF5 and UQCC1. Notably, the GWAS Catalog (date of search: February 2023) registered rs34414056 as a trait susceptibility SNP such as the waist–hip index.

Disease ontology analysis of the UK GWAS gene sets of DDH enriched the gene sets of bipolar disorder. This feature was also observed in the merged gene set, including the additional genes *IMPA1* and *DNAH8* from the Japanese GWAS. Patients with the hip disease exhibit significant pain, anxiety, and depression [49,50]. While we considered the possibility of psychiatric abnormalities secondary to hip pain, it is also possible that OA and DDH might be associated with psychiatric disorders. This study is the first to report the possibility of an association between DDH and psychiatric disorders using a genetic analysis approach. A report described a case of neurodevelopmental disorder resulting from an abnormality in Golgi-associated retrograde protein (GARP), of which *VPS53* is a component, and was described to be associated with hip dislocation [51]. Abnormalities in GARP may cause neurodevelopmental disorders and hip dislocations. This case report supports our findings, suggesting a link between DDH and mental and neurological disorders. Although further research is needed to confirm the association between DDH and psychiatric disorders, this study may elucidate the pathogenesis of intractable hip diseases.

This study had several limitations. First, the DDH cases used in the analysis were from a single institution and examined patients from a localized area in Japan (the northeastern region of Japan), and the number of cases was small. Future large-scale studies should include cases from other regions in Japan. Second, transcriptome analysis was performed on a small, limited number of cases because cartilage samples were collected from postoperative specimens, and future studies should examine larger numbers of DDH and femoral neck fracture cases and compare them with healthy cartilages.

In conclusion, using GWAS, a comparison of results from patients with DDH and those from healthy controls showed that the ferroptosis signaling pathway was associated with the disease, although no unique disease susceptibility genes were identified in Japanese patients with DDH. Transcriptome analysis of chondrocytes showed that the genes involved in the ferroptosis signaling pathway were expressed in these cells, and their expression was downregulated in the DDH-related OA group compared with that in the control group. Studies on the association between ferroptosis signaling and DDH may contribute to the search for disease susceptibility genes for DDH and to the elucidation of novel mechanisms to prevent cartilage damage.

## 4. Materials and Methods

### 4.1. Patients

The present study included 238 Japanese patients with DDH. All patients were treated at our facility and diagnosed as having DDH using radiographic images, and the history of treatment was confirmed using medical records. The inclusion criteria were hip pain and radiographic findings with a center–edge angle of less than 20°. Control data for 2044 individuals from the general Japanese population in the same region were also obtained. Written informed consent was obtained from all the participants. The study was approved by the Committee on Research Ethics of Tohoku University Graduate School of Medicine (2017-1-296), Tohoku Medical Megabank Organization (2017-0010), Kyoto University (G1208, G1216), and the National Center for Global Health and Medicine (NCGM-A-003267). All methods were performed in accordance with the institutional ethical guidelines and regulations.

Human articular cartilage samples were obtained from the femoral heads of 26 patients, collected after total hip arthroplasty for secondary hip OA associated with DDH, and from the femoral neck fracture of 22 patients as controls after hemiarthroplasty. Surgery was performed at the affiliated hospital of Tohoku University Department of Orthopedic Surgery. The study was approved by the Ethics Committee of the Tohoku University School of Medicine, and informed consent was obtained from all participating patients.

### 4.2. Genotyping, QC, Imputation, and Association Analyses

Genomic DNA was extracted from whole blood using the standard phenol-chloroform extraction–precipitation method with the PAX gene DNA Kit (BD, Franklin Lakes, NJ, USA). Genome-wide SNP genotypes of patients with DDH were determined using the Japonica v1 array (Toshiba, Tokyo, Japan), which is an SNP array specifically designed for the Japanese population. The array contained 659,253 SNPs, including tag SNPs for imputation and SNPs associated with phenotypes from previously reported GWAS and pharmacogenomics studies [52]. Genotype calling was performed using the apt-probeset-genotype program in Affymetrix Power Tools ver. 1.18.2 (Thermo Fisher Scientific Inc., Waltham, MA, USA). Sample QC was conducted according to the manufacturer’s recommendations (dish QC > 0.82 and sample call rate >97%). The clustering of each SNP was evaluated using the Ps classification function in the SNPolisher package (version 1.5.2, Thermo Fisher Scientific Inc.). SNPs that were assigned “recommended” by the Ps classification function were used for downstream analyses. Pre-phasing was conducted using EAGLE v2.3.2, with the default settings. Genotype imputation was conducted using IMPUTE4 v1.0, with a phased reference panel of Phase 1 version 3 of 1000 G containing 1092 individuals. These procedures were conducted using default settings. Cryptic relatives were excluded using PRIMUS with the default settings. Principal component (PC) analysis (PCA) was performed using East Asian samples from the International 1000 Genome Project (104 Japanese in Tokyo, 103 Han Chinese in Beijing, 93 Southern Han Chinese, 91 Chinese Dai in Xishuangbanna, and 99 Kinh in Ho Chi Minh City), in addition to the case and control samples. The PCA identified the outliers to be excluded using the Smirnov–Grubbs test with a Bonferroni-corrected *p* < 0.05.

Association analysis was performed using PLINK version 1.9. The following options were used for PLINK: call rate >97.0%; Hardy–Weinberg equilibrium *p* > 0.000001; MAF >1%; and logistic regression modeling. An association analysis was also performed with the UK Biobank dataset, with the ethnic group genetically assigned to the category of White British (Data-Field 22006). Categorized as M161, M162, or M163 in ICD-10, the DDH case samples were selected, and samples with any ICD-10 assignment were selected as the healthy controls. The demographics of the samples are presented in Table S4. 

### 4.3. Association Analysis and Gene Assignment

The GWAS was performed using PLINK version 1.9. The following options were used for PLINK: call rate >97.0%; Hardy–Weinberg equilibrium *p* > 0.000001; MAF > 1%; and logistic regression modeling with PC1 and PC2 as covariates. Four direct genotyping SNPs reached the genome-wide significance threshold (rs11802858, rs2554380, rs79657649, and rs17699467). For all four SNPs, the clustering status was “Poly high-resolution” and passed the SNP QC. To examine the misclassification of genotypes, for example, hetero as alternative homo and alternative homo as hetero, we checked the genotyping concordance rates between the genotype from the SNP array and the whole-genome sequence from the shared 190 samples. The concordance rates to whole-genome sequencing for all SNPs were consistent (Table 2), and all SNPs (rs11802858, rs2554380, rs79657649, and rs17699467) were included in the GWAS. The GWAS was also conducted on the UK Biobank data (3315 cases and 74,038 controls) using the fastGWA-GLMM [53] method of GCTA [54] with sex and PC1 to PC10 as covariates (call rate >95.0% and MAF > 1%).

Genes were assigned to SNPs with *p* < 1 × 10^−5^ in the GWAS by FUMA (https://fuma.ctglab.nl/ (accessed on 1 August 2022)) with the default settings. In the Japanese GWAS, 684 variants passed the criteria, and 42 genes were assigned. In the UK GWAS, 1622 variants passed the criteria, and 81 genes were assigned. These gene sets and merged gene sets (123 genes) were used for further GSEAs.

### 4.4. Quantitation of mRNA Expression in Chondrocytes from Patients with OA and Those with Femoral Neck Fracture

RNA extraction was performed as previously described [55,56]. Briefly, cartilage samples were cut into small pieces and suspended in the QIAzol Lysis Reagent (Qiagen, Crawley, UK). The suspension was homogenized on ice using a TissueRuptor (Qiagen, Crawley, UK) to crush the cartilage pieces and then extracted from the supernatant prepared according to the manufacturer’s instructions. RNA expression was quantified as previously described [56]. The total RNA extracted from 12 OA and eight femoral neck fracture cartilages, with RNA integrity numbers greater than 6.5, was subjected to microarray analysis using the 3D-Gene Human Oligo Chip 25k (Toray Industries, Tokyo, Japan). The extracted total RNA was labeled with Cy5 using the Amino Allyl MessageAmp II aRNA Amplification Kit (Applied Biosystems, Foster, CA, USA). The Cy5-labeled aRNA pool was applied to the hybridization buffer and hybridized for 16 h according to the supplier’s protocol. Fluorescence signals were scanned using a 3D-Gene Scanner (Toray Industries, Inc., Tokyo, Japan) and analyzed using the 3D-Gene Extraction software (Toray Industries, Inc.). A normalization method was used to adjust the median of all the detected signal intensities to 25. The mean fold change between the OA and control groups was less than 0.5, or greater than 2, and the round-robin comparison of gene expression between 12 OA and 8 control samples (96 combinations in total) showed that more than 80% (77 out of 96 combinations) of the combinations had significant differences in gene expression [56]. Genes for which significant differences were found in 80% or more of the samples (77 or more out of 96 combinations) were selected for analysis. This analysis aimed to narrow down highly specific genes without affecting mean gene expression. Results with *p* < 0.05 were considered statistically significant.

### 4.5. Gene Mapping of Disease-Related Variants and Gene Set Enrichment Analysis (GSEA)

GSEAs were performed using FUMA [57], pathway analysis in IPA (https://digitalinsights.qiagen.com/ja/qiagen-ipa/ (accessed on 1 August 2022)), and disease analysis using ToppGene (https://toppgene.cchmc.org/enrichment.jsp (accessed on 1 August 2022)), with each of the gene sets derived from the GWAS and mRNA microarray. The default parameters on the indicated website were used for all analyses.

### 4.6. Quantitative Real-Time Polymerase Chain Reaction (RT-PCR)

Qualitative RT-PCR was performed in 14 cases of DDH-related OA and 14 cases of femoral neck fracture (control), as previously described [58,59]. cDNA was synthesized from total RNA using a cDNA reverse transcription kit (Applied Biosystems, Foster City, CA, USA). Real-time amplification of target genes was performed using Taqman Universal Master Mix II containing uracil-N glycosylase and ready-to-use Taqman Gene Expression Assays (Applied Biosystems) with GTP cyclohydrolase 1 (*GCH1,* Hs 00609198_m1) and glyceraldehyde triphosphate dehydrogenase (*GAPDH*, Hs 02786624_g1) as endogenous controls. Relative gene expression data were calculated by the delta-delta-Ct method with PCR-efficiency correction using StepOne software (version 2.2.2; Applied Biosystems).

## Figures and Tables

**Table 1:** Clinical characteristics of Japanese patients with developmental dysplasia of the hip (DDH).

	n = 238
Sex (%)	206 females (86.5%)
Age (years)	60.0 ± 15.0
Positive family history of DDH (%)	94 (39.4%)
Treatment history in childhood (%)	110 (46.2%)
Surgical treatment (%)	161 (67.6%)
Total hip arthroplasty (%)	72 (30.0%)

Age is expressed as the mean ± standard deviation.

**Table 2:** Significant SNPs from Japanese GWAS and the UK Biobank GWAS. The nearest genes and eQTL genes in GTEx are shown.

(**a**) Japanese GWAS						
CHR	SNP	BP	OR	*p*	Nearest gene	GTEx eQTL gene
1	rs11802858	4682515	0.3631	1.98 × 10^−11^	*AJAP1*	NS
2	rs149003127	79236134	0.2296	1.82 × 10^−8^	*REG3G, REG1B*	NA
11	rs55669018	132871263	1.785	4.09 × 10^−8^	*OPCML*	NS
15	rs2554380	84315884	4.022	1.04 × 10^−16^	*ADAMTSL3*	*ADAMTSL3, CSPG4P11, DNM1P41, DNM1P51, GOLGA2P7, GOLGA6L4, GOLGA6L5P, LINC00933, RP11-182J1.14, RP11-671M22.4, SCAND2P, UBE2Q2L, UBE2Q2P1*
16	rs77485026	8846734	0.1884	1.34 × 10^−8^	*ABAT*	NA
17	rs79657649	505105	0.1957	1.19 × 10^−18^	*VPS53*	NS
17	rs17699467	11359275	0.351	9.82 × 10^−11^	*SHISA6*	NS
(**b**) UK Biobank GWAS						
CHR	dbSNPID	Position	Beta	*p*	Nearest gene	GTEx eQTL gene
12	rs11049197	27996732	0.203	2.62 × 10^−9^	*KLHL42*	NS

NS: Any significant eQTL association not reported in GTEx database. NA: SNP not registered in the GTEx database. Nearest gene: The nearest gene (TSS or TES) to the reported SNP. GTE: Genotype–tissue expression; QTL: Quantitative trait locus; SNP: single nucleotide polymorphism; TSS: Transcription end site; TES: Transcription start site.

**Table 3:** (**a**) Pathway enrichment analysis of 42 Japanese GWAS genes using IPA. One pathway is significant (BH FDR < 0.1 and more than three genes in the pathway category). (**b**) Disease-related gene enrichment analysis of 96 UK GWAS genes using ToppGene. Two diseases are significant (BF *p*-value < 0.01, with more than three genes in the disease category). (**c**) Pathway enrichment analysis to merge 138 genes with FUMA2FUNC. The two categories meet the condition with at least one gene derived from both cohort studies and adj*. p* < 0.05. (**d**) Pathway enrichment analysis to merge 138 genes with IPA. One pathway meets the condition with at least one gene derived from both cohort studies and a nominal *p-*value < 0.01. (**e**) Disease-related gene enrichment analysis to merge 138 genes with ToppGene. Two diseases meet the condition, with a BF *p*-value < 0.05. Only the second-highest hit passes the condition, and at least one gene is derived from both cohort studies. (**c**–**e**) Enriched genes in the Japanese and UK studies are listed in brackets after Japan and UK, respectively. All gene orders (within parentheses) are alphabetical.

(**a**)				
Ingenuity canonical pathways		Nominal *p*-value	BH FDR	Genes
Ferroptosis signaling pathway		0.0015	0.065	Japan *(CTSB, FDFT1, GCH1*)
(**b**)				
Disease category (ID)		Nominal *p*-value	BF *p*-value	Genes
Osteoarthritis of the hip (C0029410; DisGeNET curated)		1.16 × 10^−12^	2.74 × 10^−9^	UK (*IL11, MAPT, ITIH1, CRHR1, LMX1B, ASTN2, LTBP3*)
Bipolar disorder (C0005586; DisGeNET curated)		4.05 × 10^−6^	9.57 × 10^−3^	UK (*ITIH1, CRHR1, ITIH3, PDLIM5, ITIH4, STAB1, NEK4, PBRM1, MTHFD1, DLG2, ASTN2*)
(**c**)				
Category	Nominal *p*-value	Adj. *p*	Genes	
Cytoskeleton organization (GO: 0007010)	5.00 × 10^−6^	0.00296	Japan (*ARHGAP28, DNAH7, DNAH8, FAT1, FLNB, MAP6, SIGLEC15*) UK (*ABLIM1, ABLIM2, BRSK1, DNAH1, HIP1, LMOD3, MAPT, NISCH, SYNE2, SYNPO, TRIM32, TWF2*)	
Cell–cell signaling (GO: 0007267)	1.08 × 10^−6^	0.00296	Japan (*ABAT, C1QTNF3, CELF4, DGKI, FAT1, GLP1R, SHISA6*) UK (*BRSK1, CRHR1, CTBP2, DLG2, ESR2, HTR3E, IL11, MAPT, NISCH, PSEN1, STAB1, SYNPO, TMF1, WNT3*)	
(**d**)				
Ingenuity canonical pathways		*p*-value	BH FDR	Genes
Ferroptosis signaling pathway		0.006026	0.477529	Japan (*CTSB, FDFT1, GCH1*) UK (*BAP1*)
(**e**)				
Disease category (ID)		Nominal *p*-value	BF *p*-value	Genes
Osteoarthritis of the hip (C0029410; DisGeNET curated)		1.66 × 10^−11^	5.16 × 10^−8^	Japan (no hit) UK (*IL11, MAPT, ITIH1, CRHR1, LMX1B, ASTN2, LTBP3*)
Bipolar disorder (C0005586; DisGeNET curated)		5.66 × 10^−6^	1.75 × 10^−2^	Japan (*IMPA1, DNAH8*) UK (*ITIH1, CRHR1, ITIH3, PDLIM5, ITIH4, STAB1, NEK4, PBRM1, MTHFD1, DLG2, ASTN2*)

IPA: ingenuity pathway analysis; BH FDR: Benjamini–Hochberg false discovery rate; BF: Bonferroni.

## Data Availability

The summary statistics of the Japanese DDH GWAS data have been deposited in the Medical Genomics Japan Variant Database (MGeND) with the accession ID MGS000063. Raw data is available to the corresponding authors upon reasonable request.

## Supplementary Materials

The following supporting information can be downloaded at: https://www.mdpi.com/article/10.3390/ijms24055019/s1.
