# Supplementary material for: Genome-Wide Association Study and Transcriptome of Japanese Patients with Developmental Dysplasia of the Hip Demonstrates an Association with the Ferroptosis Signaling Pathway
Source: Int J Mol Sci. 2023 Mar 6;24(5):5019. doi: 10.3390/ijms24055019 (PMC10003185; doi:10.3390/ijms24055019)
Supplement: Supplementary file 1 [file ijms-24-05019-s001.zip › ijms-2198350-supplementary.pdf]

International Journal of *Molecular Sciences*

MDPI

Supplementary Material

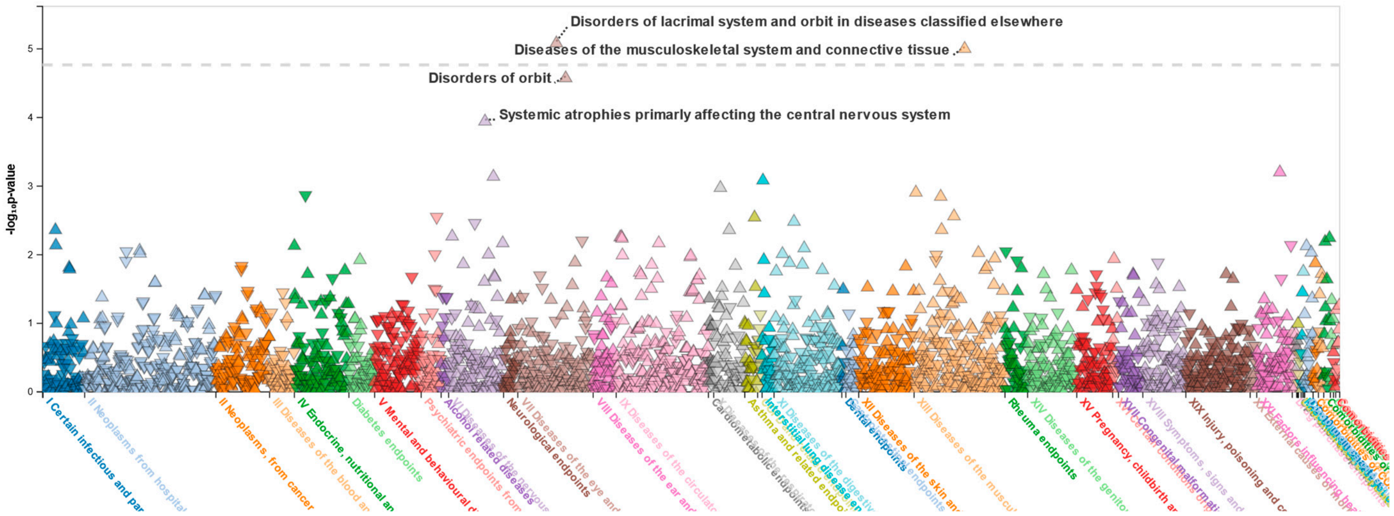

Figure S1. PheWAS of the Finnish population in the data freeze 5 to rs17699467.

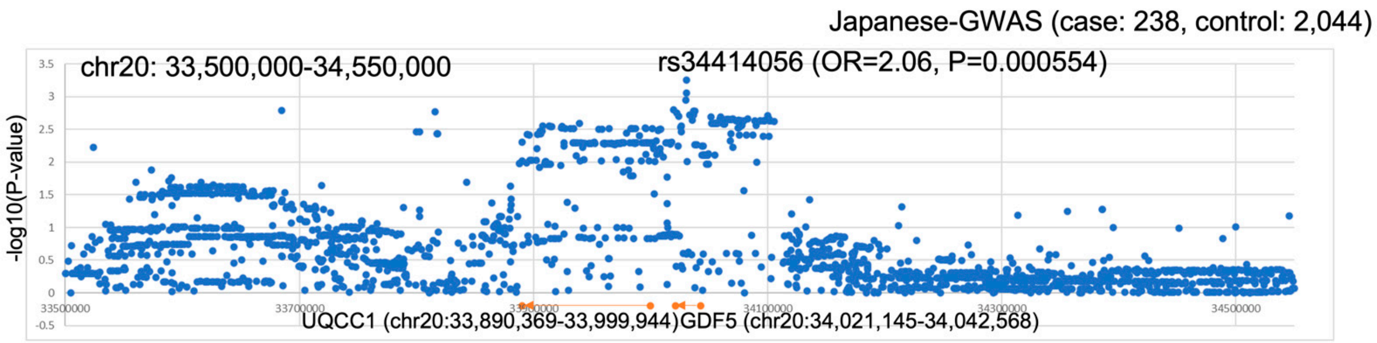

Figure S2. SNPs around GDF5-UQCC1 (Japanese population GWAS).

Table S1. Concordance rate calculation to the whole genome sequencing data

	matched	unmatched	concordance rate
rs11802858	190	0	1
rs2554380	189	1	0.99
rs79657649	190	0	1
rs17699467	190	0	1

Table S2. List of the 42 genes assigned by FUMA from Japanese GWAS

ensg	entrez	symbol
ENSG00000196482	2104	ESRRG
ENSG00000143954	130120	REG3G
ENSG00000172023	5968	REG1B
ENSG00000115386	5967	REG1A
ENSG00000118997	56171	DNAH7
ENSG00000231672	729582	DIRC3
ENSG00000079308	7145	TNS1
ENSG00000174839	201627	DENND6A
ENSG00000136068	2317	FLNB
ENSG00000145431	56034	PDGFC
ENSG00000174473	442117	GALNTL6
ENSG00000083857	2195	FAT1
ENSG00000242110	23600	AMACR
ENSG00000273294	100534612	RP11-1084J3.4
ENSG00000082196	114899	C1QTNF3
ENSG00000124721	1769	DNAH8
ENSG00000112164	2740	GLP1R
ENSG00000157680	9162	DGKI
ENSG00000079459	2222	FDFT1
ENSG00000164733	1508	CTSB
ENSG00000133731	3612	IMPA1
ENSG00000253598	347051	SLC10A5
ENSG00000155087	4956	ODF1
ENSG00000197790	119772	OR52M1
ENSG00000171533	4135	MAP6
ENSG00000183715	4978	OPCML
ENSG00000127311	92797	HELB
ENSG00000155974	23426	GRIP1
ENSG00000135119	84900	RNFT2
ENSG00000131979	2643	GCH1
ENSG00000156218	57188	ADAMTSL3
ENSG00000183044	18	ABAT
ENSG00000184857	25880	TMEM186
ENSG00000140650	5373	PMM2

ENSG00000153048	23589	CARHSP1
ENSG00000141252	55275	VPS53
ENSG00000188803	388336	SHISA6
ENSG00000088756	79822	ARHGAP28
ENSG00000101489	56853	CELF4
ENSG00000197046	284266	SIGLEC15
ENSG00000152223	57724	EPG5
ENSG00000129437	43847	KLK14

Table S3. List of the 96 genes assigned by FUMA from UKB GWAS

ENSG00000142599	473	RERE
ENSG00000074800	2023	ENO1
ENSG00000198162	10905	MAN1A2
ENSG00000092607	6913	TBX15
ENSG00000198756	23127	COLGALT2
ENSG00000068024	9759	HDAC4
ENSG00000023330	211	ALAS1
ENSG00000239732	54106	TLR9
ENSG00000173366	54106	TLR9
ENSG00000247596	11344	TWF2
ENSG00000164088	132160	PPM1M
ENSG00000164091	80335	WDR82
ENSG00000168237	132158	GLYCTK
ENSG00000114841	25981	DNAH1
ENSG00000163930	8314	BAP1
ENSG00000010318	51533	PHF7
ENSG00000010319	56920	SEMA3G
ENSG00000114854	7134	TNNC1
ENSG00000010322	11188	NISCH
ENSG00000010327	23166	STAB1
ENSG00000168268	64943	NT5DC2
ENSG00000168273	440957	SMIM4
ENSG00000163939	55193	PBRM1
ENSG00000163938	26354	GNL3
ENSG00000016864	55830	GLT8D1
ENSG00000114902	28972	SPCS1
ENSG00000114904	6787	NEK4
ENSG00000055957	3697	ITIH1
ENSG00000162267	3699	ITIH3
ENSG00000055955	3700	ITIH4
ENSG00000243696	NA	RP5-966M1.6
ENSG00000272573	389125	MUSTN1
ENSG00000248592	100526772	TMEM110-MUSTN1
ENSG00000213533	375346	TMEM110
ENSG00000163935	51460	SFMBT1

ENSG00000272305	NA	RP11-894J14.5
ENSG00000163933	91869	RFT1
ENSG00000163378	285203	EOGT
ENSG00000144747	7110	TMF1
ENSG00000163380	56203	LMOD3
ENSG00000114541	23150	FRMD4B
ENSG00000181804	285195	SLC9A9
ENSG00000186038	285242	HTR3E
ENSG00000145191	8893	EIF2B5
ENSG00000163995	84448	ABLIM2
ENSG00000163110	10611	PDLIM5
ENSG00000171604	51523	CXXC5
ENSG00000070614	3340	NDST1
ENSG00000171992	11346	SYNPO
ENSG00000154269	5169	ENPP3
ENSG00000127946	3092	HIP1
ENSG00000155158	158219	TTC39B
ENSG00000148219	23245	ASTN2
ENSG00000119401	22954	TRIM32
ENSG00000136944	4010	LMX1B
ENSG00000099204	3983	ABLIM1
ENSG00000175029	1488	CTBP2
ENSG00000173727	NA	AP000769.1
ENSG00000142186	57410	SCYL1
ENSG00000168056	4054	LTBP3
ENSG00000173465	10534	SSSCA1
ENSG00000176973	23625	FAM89B
ENSG00000173442	254102	EHBP1L1
ENSG00000137494	338699	ANKRD42
ENSG00000137500	60492	CCDC90B
ENSG00000150672	1740	DLG2
ENSG00000054654	23224	SYNE2
ENSG00000140009	2100	ESR2
ENSG00000100714	4522	MTHFD1
ENSG00000119599	26094	DCAF4
ENSG00000165861	53349	ZFYVE1
ENSG00000119707	58517	RBM25
ENSG00000080815	5663	PSEN1

ENSG00000159314	201176	ARHGAP27
ENSG00000225190	9842	PLEKHM1
ENSG00000120088	1394	CRHR1
ENSG00000185294	162540	SPPL2C
ENSG00000186868	4137	MAPT
ENSG00000256762	246744	STH
ENSG00000120071	284058	KANSL1
ENSG00000228696	100506084	ARL17B
ENSG00000176681	9884	LRRC37A
ENSG00000238083	474170	LRRC37A2
ENSG00000185829	51326	ARL17A
ENSG00000073969	4905	NSF
ENSG00000108379	7473	WNT3
ENSG00000154217	26207	PITPNC1
ENSG00000184828	201501	ZBTB7C
ENSG00000160469	84446	BRSK1
ENSG00000180061	284417	TMEM150B
ENSG00000267706	NA	CTD-2105E13.6
ENSG00000180043	284418	FAM71E2
ENSG00000095752	3589	IL11
ENSG00000160472	147744	TMEM190
ENSG00000198185	55713	ZNF334
ENSG00000149635	128506	OCSTAMP
ENSG00000182463	128553	TSHZ2

Table S4. Demographics of UK biobank samples used in this study

	DDH (n=3,315)	control (n=74,038)
Gender (%)	1987 Females (59.9%)	37,804 Females (51.1%)
Age (years)	61.9 (S.D ±6.0)	54.7 (S.D ±7.8)
